# LAMP technology: Rapid identification of *Brucella* and *Mycobacterium avium* subsp. *paratuberculosis*

**DOI:** 10.1590/S1517-838246220131206

**Published:** 2015-06-01

**Authors:** Marcos D. Trangoni, Andrea K. Gioffré, María E. Cerón Cucchi, Karina C. Caimi, Paula Ruybal, Martín J. Zumárraga, Silvio L. Cravero

**Affiliations:** 1 Instituto Nacional de Tecnología Agropecuaria, National Agricultural Technology Institute, Buenos Aires, Argentina, Institute of Biotechnology, Centre of Agronomy and Veterinary Sciences, National Agricultural Technology Institute, Buenos Aires, Argentina.; 2 Instituto Nacional de Tecnología Agropecuaria, National Agricultural Technology Institute, Buenos Aires, Argentina, Institute of Pathobiology, Centre of Agronomy and Veterinary Sciences, National Agricultural Technology Institute, Buenos Aires, Argentina.

**Keywords:** loop-mediated isothermal amplification, molecular typing, brucellosis, paratuberculosis

## Abstract

In this study, we developed new sets of primers to detect *Brucella* spp. and *M*. *avium* subsp. *paratuberculosis* (MAP) through isothermal amplification. We selected a previously well-characterized target gene, *bscp*31, specific for *Brucella* spp. and IS*900* for MAP. The limits of detection using the loop-mediated isothermal amplification (LAMP) protocols described herein were similar to those of conventional PCR targeting the same sequences. Hydroxynaphtol blue and SYBR Green^TM^ allowed direct naked-eye detection with identical sensitivity as agarose gel electrophoresis. We included the LAMP-based protocol in a rapid identification scheme of the respective pathogens, and all tested isolates were correctly identified within 2 to 3 h. In addition, both protocols were suitable for specifically identifying the respective pathogens; in the case of *Brucella*, it also allowed the identification of all the biovars tested. We conclude that LAMP is a suitable rapid molecular typing tool that could help to shorten the time required to identify insidious bacteria in low-complexity laboratories, mainly in developing countries.

## Introduction

Brucellosis and paratuberculosis are diseases caused bacterial pathogens of veterinary concern (Manning and Collins, 2001; Samartino and Enright, 1993). Brucellosis is clearly defined as a zoonotic disease. In Santa Fe province, which accounts for 32% of milk production in Argentina, the cumulative incidence rate of zoonoses in rural veterinarians (1964–2008) was reported to be 34.1%, with a brucellosis frequency of 29.1% (Molineri *et al.*, 2013). However, the role of *Mycobacterium avium* subsp. *paratuberculosis*, the causal agent of paratuberculosis, in Crohn's disease in humans is currently under discussion (Das and Seril, 2012; Kuenstner, 2012).

Brucellosis is caused by facultative intracellular pathogens of the *Brucella* genus, and domestic and wild animals are considered natural reservoirs of the disease. *Brucella melitensi*s, *Brucella abortus* and *Brucella suis* also induce human disease, and rare but persisting cases of human brucellosis caused by *Brucella canis* and *Brucella* species of marine mammals have also been recognized (Pappas *et al.*, 2005).

*M. avium* subsp. *paratuberculosis* (MAP) belongs to the *M. avium-intracellulare* complex (MAC), comprising two species, *M. intracellulare* and *M. avium*, and the subspecies *M. avium* subsp*. avium, M. avium* subsp. *hominisuiss*, *M. avium* subsp. *silvaticum* and MAP (Mijs *et al.*, 2002)*.* MAC members possess properties that enable them to grow in natural biotopes without losing their pathogenicity for certain hosts (Biet *et al.*, 2005). MAP causes chronic progressive enteritis in ruminants, which is known as paratuberculosis or Johne's disease (Chiodini *et al.*, 1984; Larsen *et al.*, 1975).

The traditional methods for detecting these pathogens are largely based on phenotypic traits, and the diagnosis of brucellosis and paratuberculosis involves bacteriological culture, histopathology and serological tests such as enzyme-linked immunosorbent assay (ELISA)-based techniques and agglutination tests (Gall *et al.*, 2008; Manning and Collins, 2001). However, the isolation of the pathogen is required to confirm the diagnosis, a process that is time consuming, especially for MAP, which requires long periods (up to two months) to develop in culture media. Molecular biology techniques have allowed the sensitive diagnosis of different bacteria through the application of nucleic acid amplification, which minimizes the requirement of biosafety conditions. In addition to contributing to the diagnosis, nucleic acid amplification provides an accurate molecular tool for identification at the species or subspecies level. The polymerase chain reaction (PCR) is the main nucleic acid amplification method currently used, and it is expected that this technique will eventually supersede many of the classical direct methods of infectious agent detection (OIE, 2008). Indeed, high sensitivity, specificity and rapidity are the major advantages of PCR over other nucleic acid-based techniques. Nonetheless, PCR requires basic equipment, such as thermocyclers, electrophoretic systems and PCR-product detection systems, and the lack of such equipment often limits its use in developing countries.

The loop-mediated isothermal amplification (LAMP) technique is characterized by its simplicity because the entire process of amplification and detection is performed in a single step in which the reaction components are subjected to isothermal conditions (Nagamine *et al.*, 2002; Notomi *et al.*, 2000), which requires less specialized equipment than conventional PCR technologies. Therefore, LAMP is accessible for laboratories in developing countries.

The LAMP method is based on the isothermal strand-displacement activity of the *Bacillus subtilis-*derived *Bst* DNA polymerase. This enzyme when combined with four target-specific primers renders the single-temperature amplification of a highly specific fragment from a DNA template at amounts greater than those of an equivalent PCR (Nagamine *et al.*, 2002; Notomi *et al.*, 2000). Furthermore, this higher amplification efficiency allows straightforward visual detection by colorimetric methods (Goto *et al.*, 2009; Parida *et al.*, 2008).

Many studies have referred to LAMP as a successful and promissory alternative for the sensitive and specific detection of human and veterinary pathogens (Barkway *et al.*, 2011; Dukes *et al.*, 2006; Savan *et al.*, 2004; Sirichaisinthop *et al.*, 2011; Wang *et al.*, 2012; Zhang *et al.*, 2013). The main objective of the present study was to develop and apply a LAMP strategy for the specific detection of *Brucella* spp. and MAP, important bacterial pathogens in Argentina, to simplify diagnosis. The purpose of this strategy focuses on lowering the reaction time and equipment costs for bacterial detection.

## Materials and Methods

### Bacterial strains and growth conditions

To standardize the LAMP protocols, we used a *B. abortus* S2308 strain and a wild-type MAP isolate, which was previously typed by conventional methods in our laboratory.

The *B. abortus* S2308 colonies were obtained from tryptose agar plates and grown in 2.5 mL tryptic soy broth (Difco BD, USA) at 200 rpm for 48 h at 37 °C.

The MAP isolate was first confirmed by insertion sequence *900* (IS*900*) and F57 PCR and then grown in 7H9 liquid medium (Difco, BD, USA) supplemented with 0.2% mycobactin J (Allied Monitor, Fayette, MO USA).

To evaluate the specificity of the LAMP assay and to test the performance of the LAMP protocol in crude lysates, we employed strains from the different hosts and sources listed in Results section. *Ochrobactrum anthropi* DNA was also evaluated as a negative control. All of the isolates belong to the INTA strain collection.

### DNA extraction from reference strain cultures and wild-type isolates

#### High-quality DNA extraction from reference strains

Chromosomal DNA of high quality was obtained to test the detection limit. DNA extraction from *B. abortus* S2308 was performed as follows*.* A 2.5 mL aliquot of culture was lysed with 30 μL of 25 mg/mL proteinase K (Promega, WI, USA) and 126 μL of 10% sodium dodecyl sulfate (SDS) for 2 h at 60 °C. The DNA was precipitated by adding 0.1 volumes of 3 M sodium acetate (pH 5.3) and 0.6 volumes of isopropanol and then removed with a sterile loop. The DNA was washed twice in 70% ethanol and suspended in 600 μL of Tris-EDTA (TE) buffer (10 mM Tris-HCl pH 8, 1 mM EDTA). A second step of precipitation and purification was performed. Finally, the DNA was suspended in 100 μL of TE. DNA extraction from the MAP culture was performed as previously described (van Embden *et al.*, 1992)*.* The DNA integrity was assessed by 0.8% agarose gel electrophoresis and then quantitated using a Nanodrop ND-1000 spectrophotometer (Thermo Fisher Scientific, DE, USA). Ten-fold serial DNA dilutions were performed with sterile distilled water, and 1 μL of each dilution was used as the template for amplification.

#### Rapid DNA extraction from wild-type isolates

Cell lysis was performed by physical treatment. A loopful of growth from a solid medium or a 0.5–1 mL aliquot devoid of medium by centrifugation was suspended in 200 μL of distilled water. The sample was boiled and subsequently frozen twice for 10 min each. Finally, the sample was subjected to a brief centrifugation at 12,000 × *g*, and 5 μL of the supernatant was used as the template for amplification.

### Target genes and primer design

Target sequences that are traditionally used to identify *Brucella* spp. and MAP were selected. The LAMP primers were designed to target the *bscp*31 gene from *Brucella* spp. (Baily *et al.*, 1992) and IS*900* from MAP (Green *et al.*, 1989). Complete LAMP primer sets, including both loop primers for each selected sequence, were designed using Primer Explorer V4 software (http://primerexplorer.jp/elamp4.0.0/index.html). The primer sequences are listed in Table 1.

**Table 1:** LAMP primers designed in this study.

Target organism (Protocol)	Target sequence	Primer	Sequence (5′ to 3′)	Length	LAMP T (°C)
*Brucella* spp. (Bru-LAMP)	*bcsp*31	F3-Bru	CAGACGTTGCCTATTGGGC	19-mer	60
		B3-Bru	GGCTCATCCAGCGAAACG	18-mer	
		FIP-Bru	CGGGTAAAGCGTCGCCAGAAGTTTT-GCACCGGCCTTTATGATGG	44-mer	
		BIP-Bru	ACGATCCATATCGTTGCGCGTTTTT-GCTTGCCTTTCAGGTCTGC	44-mer	
		LF-Bru	CGCAAATCTTCCACCTTGCC	20-mer	
		LR-Bru	GGATGCAAACATCAAATCGGTC	22-mer	
*M. avium* subsp. *paratuberculosis* (MAP-LAMP)	IS*900*	F3-MAP	CGCAACGCCGATACCGT	17-mer	65
		B3-MAP	CCCAGGATGACGCCGAA	17-mer	
		FIP-MAP	CATCACCTCCTTGGCCAGGC-CCGCTAACGCCCAACAC	37-mer	
		BIP-MAP	GCGACACCGACGCGATGAT-TCCGGGCATGCTCAGGA	36-mer	
		LF-MAP	AGTGGCCGCCAGTTGTTG	18-mer	
		LR-MAP	ACCGCCACGCCGAAATC	17-mer	

### LAMP reaction and detection

LAMP assays for *Brucella* spp. and MAP (Bru-LAMP and MAP-LAMP, respectively) were performed in a final reaction volume of 25 μL with 1.4 mM dNTPs (Promega, WI, USA), 8 mM SO_4_Mg, 0.8 M betaine (Sigma-Aldrich, MO, USA) and 8 U of *Bst* DNA Polymerase (New England Biolabs, MA, USA). The LAMP reactions also contained 1.6 μM of FIP and BIP primers, 0.16 μM of F3 and B3 primers, 0.8 μM of LF and LR primers and the corresponding DNA as the template. The templates consisted of 1 μL of high-quality DNA or 5 μL of the supernatant of the cell lysate obtained by the rapid DNA extraction method. The reaction tubes were incubated in different equipment (MyCycler thermocycler (Bio-Rad, CA, USA) and a thermal bath) to evaluate the robustness of the amplification method. For the MyCycler thermocycler, the tubes were incubated for 60 min at 60 °C and then for 5 min at 80 °C. The incubation using the thermal bath was for 60 min at 60 °C. For MAP-LAMP, the incubation temperature was 65 °C instead of 60 °C due to the characteristic high GC content of mycobacterial genomes. The LAMP amplicons were visualized by different strategies: a) 2% agarose gel electrophoresis, staining with 0.5 μg/mL of ethidium bromide and visualization under ultraviolet (UV) light; b) naked-eye inspection by colorimetric methods such as SYBR Green^TM^ staining and hydroxy naphthol blue (HNB). For SYBR Green^TM^ staining, 1 μL of 1/10 SYBR Green^TM^ I (Invitrogen, CA, USA) solution was added directly to each reaction tube after incubation, and the DNA was visualized under UV light. For HNB (JT Baker, USA) staining, a final concentration of 120 μM was utilized. HNB was added prior to amplification (Goto *et al.*, 2009).

### PCR amplification

The reactions were performed with primers B4/B5 for *Brucella* spp. (B4/B5 PCR) and S204/S749 for MAP (IS*900* PCR), as previously described (Baily *et al.*, 1992; Englund *et al.*, 1999). The amplification reactions were performed in a final volume of 25 μL with 1.25 U of Taq DNA Polymerase (Promega, WI, USA), 200 μM dNTPs (Promega, WI, USA), 0.5 μM of each primer and 1 μL of high-quality DNA or 5 μL of the supernatant of the cell lysates obtained during the rapid DNA extraction method. The reactions consisted of an initial denaturation step at 95 °C for 5 min, followed by 35 amplification cycles and a final extension step at 72 °C for 10 min. The amplification cycles comprised a first step at 94 °C for 1 min, an annealing step at 60 °C for *Brucella* spp. or 59 °C for MAP for 1 min and an extension step at 72 °C for 1 min. The PCRs were performed in a MyCycler thermocycler (Bio-Rad, CA, USA). The sizes of the PCR products (223 bp and 563 bp, respectively) were determined by comparison with a molecular weight marker using 1.5% agarose gel electrophoresis, ethidium bromide staining (0.5 μg/mL) and UV light visualization.

## Results

### Detection limit and specificity of LAMP *vs.* PCR

**Figure 1:**
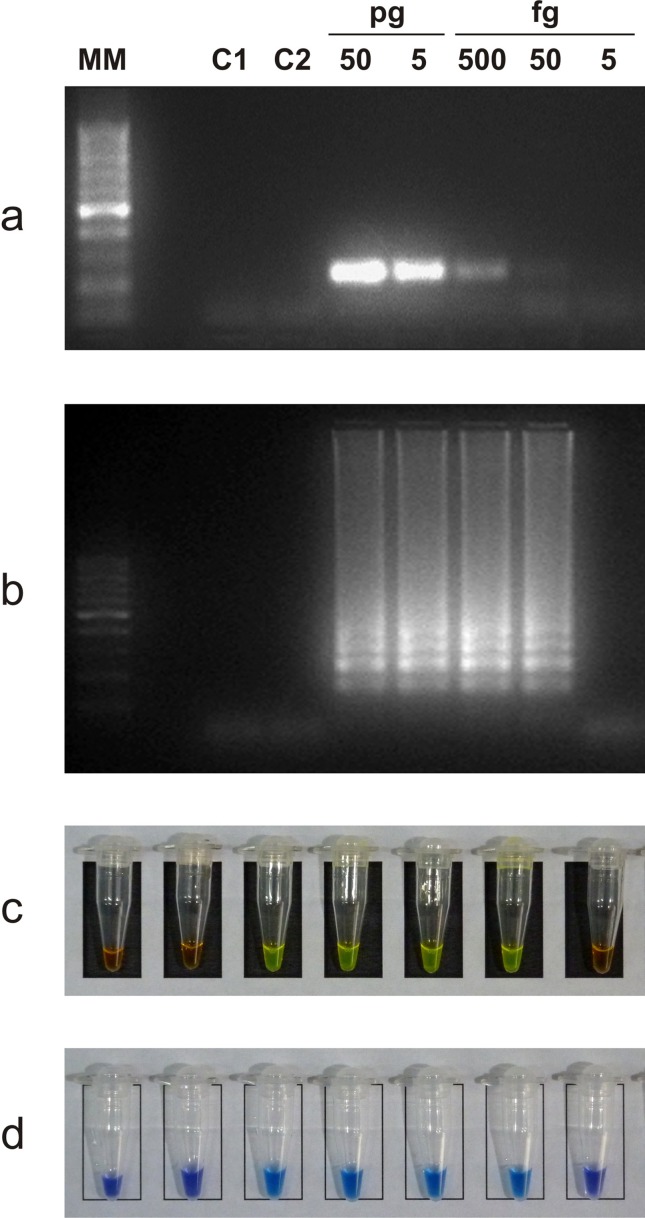
Comparative analytical sensitivity of Bru-LAMP and PCR. Agarose gel electrophoresis and direct naked-eye detection. 1a) B4/B5 PCR, 1.5% agarose gel. 1b) Bru-LAMP, 2% agarose gel. 1c) Bru-LAMP SYBR Green^TM^. 1d) Bru-LAMP, HNB. Serial DNA dilution of *Brucella abortus* S2308 (from 50 pg to 5 fg); C1, *Ochrobactrum anthropi* DNA; C2, internal PCR/LAMP negative control (water). MM: 100 bp molecular marker (Promega).

**Figure 2:**
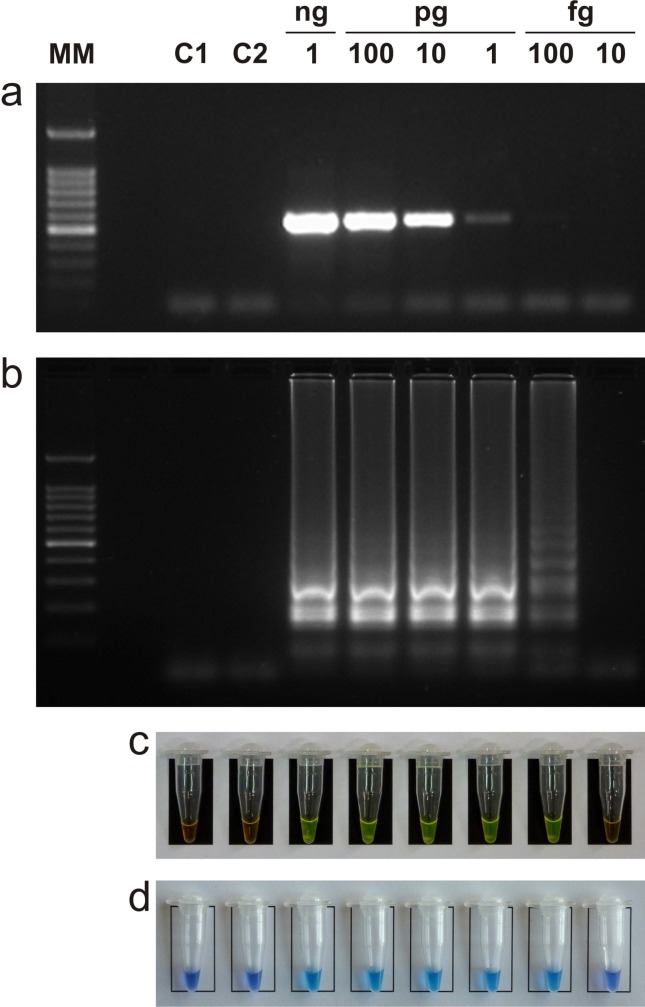
Comparative analytical sensitivity of MAP-LAMP and PCR. Agarose gel electrophoresis and direct naked-eye detection. 1a) IS*900* PCR, 1.5% agarose gel. 1b) MAP-LAMP, 2% agarose gel. 1c) MAP-LAMP, SYBR Green^TM^. 1d) MAP-LAMP, HNB. Serial DNA dilution of *M. avium* subsp. *paratuberculosis* (from 1 ng to 10 fg); C1, *M. avium* subsp. *avium* DNA; C2, internal PCR/LAMP negative control (water). MM: 100 bp molecular marker (Promega).

After confirming amplification using the novel sets of primers, we determined the detection limit by 10-fold serial dilutions of purified genomic DNA. Positive LAMP reactions were confirmed by the appearance of a ladder-like pattern on agarose gels stained with ethidium bromide; positive PCRs were confirmed by specific size amplicon visualization. The detection limit of Bru-LAMP and MAP-LAMP were 50 fg and 100 fg per reaction, respectively (Figures 1b and 2b). The sensitivities reached by all the LAMP protocols were in accordance with those obtained with PCR targeting the same genes; however, the last point detected by PCR was barely observed (Figures 1a and 2a).

The specificity of the Bru-LAMP protocol was determined using DNA from *Ochrobactrum anthropi*, a phylogenetically related bacterium, as the control. No evidence of cross-reactivity was detected with the control DNA tested by PCR (Figure 1a) or LAMP (Figure 1b).

The specificity of the MAP-LAMP protocol was evaluated using cell lysates from *M. bovis, M. avium* subsp*. avium, M. gordonae, M. scrofulaceum, M. porcinum,* and two phylogenetically related bacteria, *Nocardia farcinica* and *Nocardia testacea*. Interestingly, a specific amplification of MAP was obtained when using the MAP-LAMP protocol designed in this study (Table 2).

**Table 2:** LAMP performance and specificity evaluated using cell lysate samples from cultures of different bacteria.

Bacteria (number of isolates tested)^a^	Host/source	PCR^b^	LAMP^c^
*M. avium* subsp. *paratuberculosis* (16)	cattle sheep	+	+
*M. avium* subsp. *avium* (4)	cattle, dog, swine and human	−	−
*M. bovis* (5)	cattle	−	−
*M. gordonae* types 2, 3, 9 (3)	water	−	−
*M. scrofulaceum* (1)	human	−	−
*M. porcinum* (1)	cattle	−	−
*Nocardia farcinica* (1) *Nocardia testacea* (1)	cattle	−	−
*B. abortus* biovars 1, 2, 3, 4, 5, 6, 9 (7)	reference strains	+	+
*B. melitensis* biovars 1, 2, 3 (3)	reference strains	+	+
*B. suis* biovars 1, 2, 3, 4, 5 (5)	reference strains	+	+
*B. ovis* (REO 198 strain) (1)	reference strain	+	+
*B. canis* (RM6/66 strain) (1)	reference strain	+	+
*B. cetaceae* (1)	reference strain	+	+
*B. pinnipedialis* (1)	reference strain	+	+
*B. abortus* biovar 1 (7)	cattle, human	+	+
*B. melitensis* biovar 1 (7)	goat, human	+	+
*B. suis* biovar 1 (5)	Swine, human	+	+
*B. cetaceae* (1)	whale	+	+

a 71 samples were evaluated.

b *Mycobacterium* spp. and *Nocardia* spp. isolates were processed by IS*900* PCR. *Brucella* spp. were processed by B4/B5 PCR.

c Samples were processed according to the corresponding LAMP protocols. The end-point was evaluated by SYBR Green^TM^ (naked-eye). The same results were obtained by UV visualization.

### End-point detection of LAMP products by single staining

The end-point detection of the products of LAMP amplification was performed by naked-eye inspection by fluorescent staining for nucleic acid detection. For SYBR Green^TM^, a dilution of the original orange color indicates a negative result, whereas a fluorescent green color indicates a positive amplification. For HNB, a violet or sky-blue color indicates a negative or a positive result, respectively. All the direct end-point detections showed the same sensitivity (Figures 1c–d and 2c–d).

### Usefulness of LAMP in molecular typing schemes

**Figure 3:**
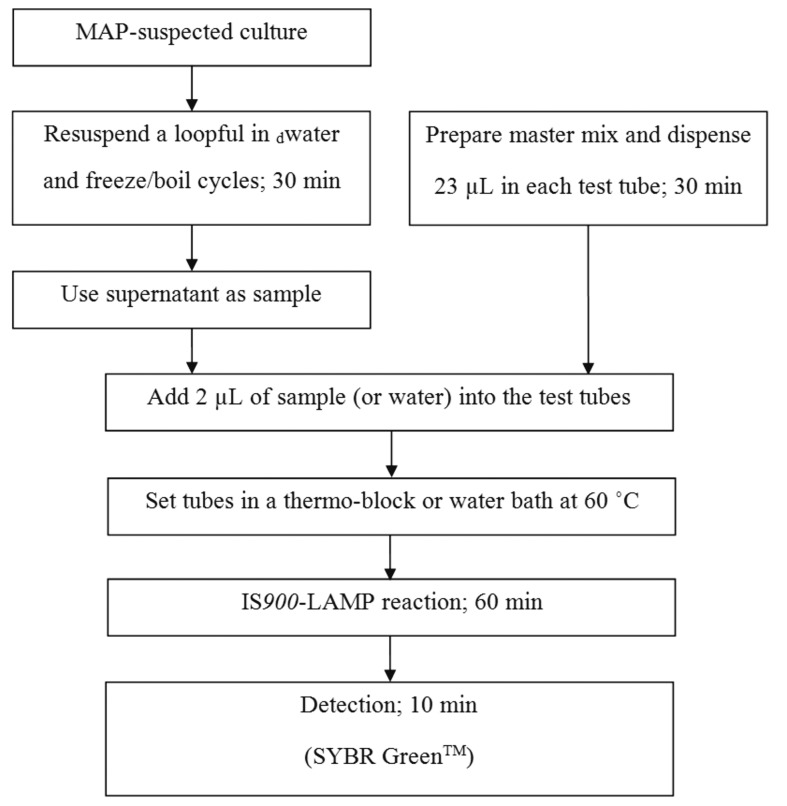
A proposed workflow for rapid identification of microorganisms through LAMP: Identification of MAP as an example. The estimated times are relative to the sample number.

Next, we evaluated the potential of LAMP as part of a rapid molecular typing scheme using non-purified DNA as the template. For this purpose, we tested 16 MAP field isolates and 20 *Brucella* spp. isolates along with 17 reference strains. As an example, Figure 3 shows how the MAP cultures were processed.

All the samples tested by the MAP-LAMP protocol were identified as MAP according to IS*900* PCR (Table 2). Additionally, cell lysates of *Nocardia* spp. and *M. avium* subsp. *avium*, an atypical mycobacteria, were also assessed; as expected, the results were negative (Table 2). The results obtained for all the samples were the same, regardless of the equipment employed (thermostatic water bath or thermocycler) (data not shown). The same protocol was adapted for *Brucella*, and the correct identification of the genus was achieved, regardless of the species or serovar tested (Table 2). Thus, a correct identification of the isolate could be obtained in less than 3 h, which demonstrates the suitability of applying LAMP as a routine protocol.

## Discussion

We aimed to develop LAMP-based protocols for the specific and sensitive detection of two bacterial pathogens. To this end, we designed primers and tested LAMP sensitivity using three different methods for end-point determination: agarose gel electrophoresis, SYBR Green^TM^ and hydroxynaphthol blue.

In our study, we specifically designed LAMP primers for the identification of *Brucella* spp. using *bcsp*31 as the target sequence. This gene encodes the 31 kDa *Brucella* cell surface salt-extractable protein (BCSP) and is highly conserved in the genus *Brucella* (Baily *et al.*, 1992; Bricker, 2002). Ohtsuki *et al.* (2008) first reported the identification of *Brucella* spp. using two LAMP primer sets targeting the same gene. The detection limit obtained in our study with the *Brucella* genus-specific LAMP was similar to that obtained by Ohtsuki *et al.* (2008). Another LAMP for the identification of members of this genus with a higher sensitivity has been previously described; however, a different gene, which codes for the 25 kDa outer membrane protein (Omp25), was selected as the target (Lin *et al.*, 2011).

Primer design is the most important factor affecting the performance of LAMP. However, further optimization of the protocol may be needed to improve the sensitivity of the test. Taking into account that the LAMP reaction combines 6 to 8 different regions, a highly specific amplification product is expected.

We obtained a detection limit with the MAP-LAMP protocol that was similar to that obtained by Enosawa *et al.* (2003), who targeted the same sequence. In the present study, we tested the specificity of the MAP primer set with DNA from closely related mycobacteria, such as *M. avium* subsp. *avium*, or *M. bovis*, another important mycobacterial pathogen of veterinary concern. Although the MAP-LAMP developed in our study proved to be discriminative for subspecies determination, some nonspecific amplification with *M. scrofulaceum* strains has been reported using LAMP targeting IS*900* (Enosawa *et al.*, 2003). We herein demonstrated that the MAP-LAMP protocol was suitable, even for a panel of atypical mycobacteria, including *M. scrofulaceum*, or *Nocardia* spp., a closely related genus.

The comparative results between the PCR and novel LAMP protocols reported here demonstrate that isothermal amplification can achieve the same sensitivity as conventional PCR, regardless of the pathogen. This is consistent with previous comparative analyses in which LAMP reached the same sensitivity, or even higher levels, as nested-PCR and real-time PCR (Enosawa *et al.*, 2003; Lin *et al.*, 2009). Hence, the high processivity of the isothermal amplification yields a detectable product faster than conventional PCR, which is a hallmark of this method.

It is important to note that the methodology used for product detection could bias the sensitivity. The PCR detection limit is established by the presence of specific bands on agarose gel electrophoresis, which depends not only on the concentration of the amplicon obtained but on several other factors. For instance, the concentration of ethidium bromide, the sensitivity of the detection system and image processing (if available), among other factors, may alter the results. However, as we showed in this study, the threshold at which the LAMP reaction changes from positive to negative is abrupt and, as a consequence, the end-point determination is accurate.

LAMP not only leads to the isothermal amplification of DNA in a stoichiometric reaction (Notomi *et al.*, 2000) but also to the variation of by-products. For instance, the increased formation of magnesium pyrophosphate (Mori *et al.*, 2001) and the subsequent reduction in the concentration of magnesium cations can be titrated by HNB (Goto *et al.*, 2009). Although the visualization of magnesium pyrophosphate precipitate is a simple end-point detection strategy (Mori *et al.*, 2001), the sensitivity of this technique is lower than that of fluorometric and colorimetric methods and often requires the use of a centrifugation step to facilitate the visualization of the precipitate or trained technicians.

Considering that an ideal detection method must fit certain criteria, such as sensitivity, reproducibility and an accessible cost, it is important to know the performance of the different methods available in the laboratory. As the use of SYBR Green^TM^ or HNB shows better reproducibility, we selected this method to compare the relative sensitivity to that of agarose gel electrophoresis. The comparative sensitivity achieved by both methods was similar in all the LAMP protocols tested. The direct determination of the end-point, however, was easier when the SYBR Green^TM^ method was performed, even without an UV-transilluminator. Although a method of choice, SYBR Green^TM^ requires the opening of the reaction tubes after amplification; which can result in carry-over contamination, and the risk of amplicon contamination limits its application to those laboratory settings in which LAMP is used as a non-routine practice. A lower color contrast between positive and negative amplification was observed when the HNB method was used; however, the sensitivity obtained in each assay was the same as the other studied methods. Therefore, the HNB method is more suitable for laboratories in which LAMP is frequently used due to its low risk of contamination.

To achieve a scheme that allows the rapid identification of bacterial pathogens, we coupled the LAMP protocol to a simple step of cell lysis that enabled us to easily release DNA from cultures. This approach allowed a significant reduction in the time required for identification compared to the traditionally time-consuming protocols that involve DNA extraction and conventional PCR. Importantly, the reduction in the complexity of the protocols could also help to expand the use of molecular biology techniques to laboratories that have not yet adopted DNA-based techniques.

The evaluation of pathogen detection directly from clinical samples certainly constitutes a challenge, and a LAMP method tested using a wide panel of field samples could be a useful tool to diagnose diseases that impact production systems. Finally, LAMP is a simple, rapid, low-cost genetic testing technology that is specific and sensitive. This technology can be coupled to schemes with typing purposes and could contribute to the conventional methods used for the identification of *Brucella* spp. and MAP in non-sophisticated laboratories, especially in developing countries.

## References

[B01] Baily GG, Krahn JB, Drasar BS (1992). Detection of *Brucella melitensis* and
*Brucella abortus* by DNA amplification. J Trop Med Hyg.

[B02] Barkway CP, Pocock RL, Vrba V (2011). Loop-mediated isothermal amplification (LAMP) assays for the
species-specific detection of Eimeria that infect chickens. BMC Vet Res.

[B03] Biet F, Boschiroli ML, Thorel MF (2005). Zoonotic aspects of *Mycobacterium bovis* and
*Mycobacterium avium*-*intracellulare*
complex (MAC). Vet Res.

[B04] Bricker BJ (2002). PCR as a diagnostic tool for brucellosis. Veterinary Microbiol.

[B05] Chiodini RJ, Van Kruiningen HJ, Merkal RS (1984). Ruminant paratuberculosis (Johne's disease): the current status
and future prospects. Cornell Vet.

[B06] Das KM, Seril DN (2012). *Mycobacterium avium* subspecies
*paratuberculosis* in Crohn's disease: the puzzle
continues. J Clin Gastroenterol.

[B07] Dukes JP, King DP, Alexandersen S (2006). Novel reverse transcription loop-mediated isothermal
amplification for rapid detection of foot-and-mouth disease
virus. Arch Virol.

[B08] Englund S, Ballagi-Pordany A, Bolske G (1999). Single PCR and nested PCR with a mimic molecule for detection of
*Mycobacterium avium* subsp.
*paratuberculosis*. Diagn Microbiol Infect Dis.

[B09] Enosawa M, Kageyama S, Sawai K (2003). Use of loop-mediated isothermal amplification of the
IS*900* sequence for rapid detection of cultured
*Mycobacterium avium* subsp.
*paratuberculosis*. J Clin Microbiol.

[B10] Gall D, Nielsen K, Nicola A (2008). A proficiency testing method for detecting antibodies against
*Brucella abortus* in quantitative and qualitative
serological tests. Rev Sci Tech.

[B11] Goto M, Honda E, Ogura A (2009). Colorimetric detection of loop-mediated isothermal amplification
reaction by using hydroxy naphthol blue. Bio Techniques.

[B12] Green EP, Tizard ML, Moss MT (1989). Sequence and characteristics of IS*900*, an
insertion element identified in a human Crohn's disease isolate of
*Mycobacterium paratuberculosis*. Nucleic Acids Res.

[B13] Kuenstner JT (2012). *Mycobacterium avium paratuberculosis* and Crohn's
Disease: an association requiring more research. J Crohn's Colitis.

[B14] Larsen AB, Merkal RS, Cutlip RC (1975). Age of cattle as related to resistance to infection with
*Mycobacterium paratuberculosis*. Am J Vet Res.

[B15] Lin GZ, Zheng FY, Zhou JZ (2011). Loop-mediated isothermal amplification assay targeting the
*omp*25 gene for rapid detection of
*Brucella* spp. Mol Cell Probes.

[B16] Manning EJ, Collins MT (2001). *Mycobacterium avium* subsp.
*paratuberculosis*: pathogen, pathogenesis and
diagnosis. Rev Sci Tech.

[B17] Mijs W, de Haas P, Rossau R (2002). Molecular evidence to support a proposal to reserve the
designation *Mycobacterium avium* subsp.
*avium* for bird-type isolates and ‘*M.
avium* subsp. *hominissuis*‘ for the
human/porcine type of *M. avium*. Int J Syst Evol Microbiol.

[B18] Molineri A, Signorini ML, Perez L (2013). Zoonoses in rural veterinarians in the central region of
Argentina. Aust J Rural Health.

[B19] Mori Y, Nagamine K, Tomita N (2001). Detection of loop-mediated isothermal amplification reaction by
turbidity derived from magnesium pyrophosphate formation. Biochem Biophys Res Commun.

[B20] Nagamine K, Hase T, Notomi T (2002). Accelerated reaction by loop-mediated isothermal amplification
using loop primers. Mol Cell Probes.

[B21] Notomi T, Okayama H, Masubuchi H (2000). Loop-mediated isothermal amplification of DNA. Nucleic Acids Res.

[B22] Ohtsuki R, Kawamoto K, Kato Y (2008). Rapid detection of *Brucella* spp. by the
loop-mediated isothermal amplification method. J Appl Microbiol.

[B23] OIE, Commission OBS (2008). Validation and quality control of PCR methods for the diagnosis
of infectious disease. Manual of diagnostic tests and vaccines for terrestrial animals.

[B24] Pappas G, Akritidis N, Bosilkovski M (2005). Brucellosis. N Engl J Med.

[B25] Parida M, Sannarangaiah S, Dash PK (2008). Loop mediated isothermal amplification (LAMP): a new generation
of innovative gene amplification technique; perspectives in clinical
diagnosis of infectious diseases. Rev Med Virol.

[B26] Samartino LE, Enright FM (1993). Pathogenesis of abortion of bovine brucellosis. Comp Immunol Microbiol Infect Dis.

[B27] Savan R, Igarashi A, Matsuoka S (2004). Sensitive and rapid detection of edwardsiellosis in fish by a
loop-mediated isothermal amplification method. Appl Environ Microbiol.

[B28] Sirichaisinthop J, Buates S, Watanabe R (2011). Evaluation of loop-mediated isothermal amplification (LAMP) for
malaria diagnosis in a field setting. Am J Trop Med Hyg.

[B29] van Embden JD, van Soolingen D, Small PM (1992). Genetic markers for the epidemiology of
tuberculosis. Res Microbiol.

[B30] Wang F, Jiang L, Ge B (2012). Loop-mediated isothermal amplification assays for detecting shiga
toxin-producing *Escherichia coli* in ground beef and human
stools. J Clin Microbiol.

[B31] Zhang W, Meng X, Wang J (2013). Sensitive and rapid detection of *Trueperella
pyogenes* using loop-mediated isothermal amplification
method. J Microbiol Methods.

